# Neandertals Likely Kept Their Genes to Themselves

**DOI:** 10.1371/journal.pbio.0020080

**Published:** 2004-03-16

## Abstract

xx

Scientists searching for clues to our origins have long relied on studying fossils to piece together our evolutionary history. Now, with the tools of molecular genetics, they can reach beyond morphological evidence to retrieve traces of DNA preserved in the remnants of bone. And in these ancient DNA sequences, they're finding bits and pieces of the evolutionary record. Over the course of evolution, changes in DNA sequences accumulate at a predictable rate. These mutations can reveal not only how closely related we are but also when evolutionary lineages diverged. Identifying both a typical range of genetic variation and rate of mutation for a given species or population, for example, can serve as a frame of reference for analyzing DNA sequences from other species or populations. Most molecular anthropologists use DNA found in mitochondria—intracellular structures that convert food into energy—to reconstruct human evolution. Distinct from nuclear DNA, mitochondrial DNA (mtDNA) exists in the cytoplasm of a fertilized egg and is passed on only through the maternal lineage.

An ongoing debate about human origins has revolved around the theory that Homo sapiens and Homo Neanderthalensis interbred, since the two species coexisted. Neandertals lived roughly 150,000 to 30,000 years ago, toward the end of the Pleistocene era, and inhabited Europe, parts of Asia, and the Middle East. Modern-day humans arose between 100,000 and 200,000 years ago. Recently, an international multidisciplinary team of scientists led by Svante Pbo of the Max Planck Institute for Evolutionary Anthropology have analyzed the largest sample of Neandertal and early human remains to date and conclude that Neandertals could not have made a significant genetic contribution to early modern humans.

Part of the challenge of resolving the human–Neandertal interbreeding issue stems from the fact that so many fossil samples—of both early humans and more archaic humans—are contaminated with the DNA of the contemporary humans who have handled them. So even if a Neandertal sample contained a “real” (or endogenous) DNA sequence resembling early humans—which would indicate intimacy between the two groups—it might be considered contaminated. When Pääbo and colleagues looked for modern DNA, they found it in every sample they examined: in the Neandertal and early human fossils—and even in cave bear teeth. To circumvent this problem, they looked only for Neandertal mtDNA as evidence of interbreeding. Since it is easy to distinguish modern human mtDNA sequences from the four Neandertal mtDNA samples that have been sequenced so far, the researchers decided to determine whether Neandertal-like mtDNA could be found in other Neandertal fossils as well as in early human remains.[Fig pbio-0020080-g001]

**Figure pbio-0020080-g001:**
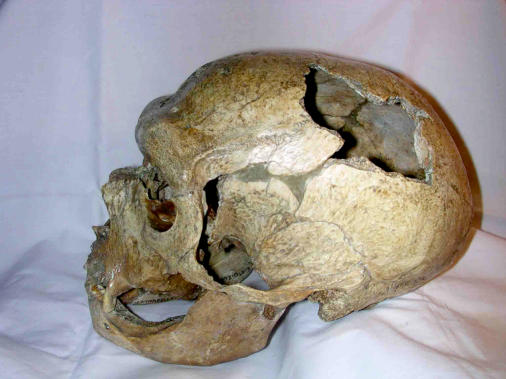
Neandertal skull from La Chapelle aux Saints

As these fossils are precious commodities, Pbo's group applied a technique developed in their lab that uses amino acid content as a measure of extractable endogenous DNA and requires removing just 10 mg of bone from a specimen rather than much larger pieces of bone. Of 24 Neandertal and 40 early modern human fossils analyzed, they found four Neandertal and five early human specimens that passed the amino acid test. These fossils included samples classified as “transitional” between the two groups and represented a wide distribution across Europe, where the two groups would likely have encountered one another. When they analyzed these samples for Neandertal mtDNA, they found mtDNA sequences that are absent in contemporary human mtDNA genes but quite similar to those found in the four previously sequenced Neandertals. They found no Neandertal-like mtDNA in the early human samples.

While the authors explain that it's impossible to definitively conclude that no genetic flow occurred between early humans and Neandertals given the limited number of early human fossils available, they point out that even fossil samples considered as anatomically transitional between modern humans and Neandertals failed to show evidence of mtDNA exchange. Thus, Pääbo and colleagues conclude, while it's possible that Neandertals made a small contribution to the genetic makeup of contemporary humans, the evidence cannot support the possibility of a large contribution.

